# Effectiveness and tolerability of camrelizumab combined with molecular targeted therapy for patients with unresectable or advanced HCC

**DOI:** 10.1007/s00262-023-03404-8

**Published:** 2023-02-25

**Authors:** Ting Li, Jiang Guo, Yushen Liu, Zhaoqing Du, Zhaoyang Guo, Yangwei Fan, Long Cheng, Yue Zhang, Xu Gao, Yunyu Zhao, Xinyuan He, Wenhua Wu, Ning Gao, Yinying Wu, Jie Li, Yu Zhang, Wen Kang, Zhifang Cai, Wenjun Wang, Xiaopeng Li, Ying Zan, Mindie H. Nguyen, Fanpu Ji

**Affiliations:** 1 Department of Infectious Diseases, The Second Affiliated Hospital of Xi’an Jiaotong University, No. 157 Xi Wu Road, Xi’an, 710004 Shaanxi Province China; 2 Department of Oncology Interventional Radiology, Beijing Ditan Hospital, Capital Medical University, Beijing, 100015 China; 3 Department of Infectious Diseases, Tangdu Hospital, the Fourth Military Medical University, Xi’an, 710038 China; 4 Department of Hepatobiliary Surgery, Shaanxi Provincial People’s Hospital, Xi’an, 710068 China; 5 Department of Infectious Disease, Shandong Provincial Hospital, Shandong University, Jinan, 250021 Shandong China; 6 Department of Medical Oncology, The First Affiliated Hospital of Xi’an Jiaotong University, Xi’an, Shaanxi China; 7 Department of Infectious Diseases, Nanjing Drum Tower Hospital, The Affiliated Hospital of Nanjing University Medical School, Nanjing, 210008 China; 8 Department of Ultrasound, The Second Affiliated Hospital of Xi’an Jiaotong University, Xi’an, 710004 China; 9 Department of Oncology, The Second Affiliated Hospital of Xi’an Jiaotong University, No.157 Xi Wu Road, Xi’an, 710004 Shaanxi Province China; 10 Division of Gastroenterology and Hepatology, Stanford University Medical Center, 750 Welch Road, Suite 210, Palo Alto, CA 94304 USA; 11 Department of Epidemiology and Population Health, Stanford University, Palo Alto, CA USA; 12 National and Local Joint Engineering Research Center of Biodiagnosis and Biotherapy, The Second Affiliated Hospital of Xi’an Jiaotong University, Xi’an, China; 13 Key Laboratory of Environment and Genes Related to Diseases, Xi’an Jiaotong University, Ministry of Education of China, Xi’an, China

**Keywords:** Hepatocellular carcinoma, Camrelizumab, Programmed cell death protein-1, Child-Pugh B, Effectiveness, Tolerability

## Abstract

There is a lack of effective programmed cell death protein 1 (PD-1)‐targeted immunotherapy with good tolerability in patients with advanced hepatocellular carcinoma (HCC) and severely compromised liver function. We assessed patient outcomes after combined camrelizumab and molecular targeted therapy in a multicenter cohort study in China. The study included 99 patients with advanced HCC (58 Child-Pugh A and 41 Child-Pugh B), 84 of them received camrelizumab combined with molecular targeted therapy from January 10, 2019, to March 31, 2021. Overall survival (OS), progression-free survival (PFS), objective response rate (ORR), disease control rate (DCR), and adverse events (AEs) were assessed. The median follow-up was 12.1 months. For patients with Child-Pugh B, the OS probability at 12-months, ORR and DCR were 49.7%, 31.7% and 65.9%, respectively, and the median PFS was 5.1 months [95% confidence interval (CI) 3.0–7.1], which were comparable with Child-Pugh A patients, although median OS was shorter in Child‐Pugh B patients (20.5 vs.13.4 months, *P* = 0.12). In multivariate analysis, macrovascular infiltration (MVI), but not sex, age, hepatitis B virus etiology, extrahepatic metastasis, Child-Pugh B, or AFP > 400 ng/ml, was associated with 12-months OS [hazard ratio (HR) 2.970, 95% CI 1.276–6.917, *P* = 0.012] and ORR (HR 2.906, 95% CI 1.18–7.16, *P* = 0.020). Grade 3/4 immune-related AEs occurred in 26.8% of Child-Pugh B patients, including one potentially treatment-related death. In both groups, the most common AEs were immune thrombocytopenia and hepatotoxicity. Camrelizumab combined with targeted therapy showed favorable effectiveness and tolerability with manageable toxicities in Chinese HCC patients, regardless of Child-Pugh A/B liver function. MVI was associated with suboptimal immunotherapy response and poor prognosis.

**Graphical abstract:**

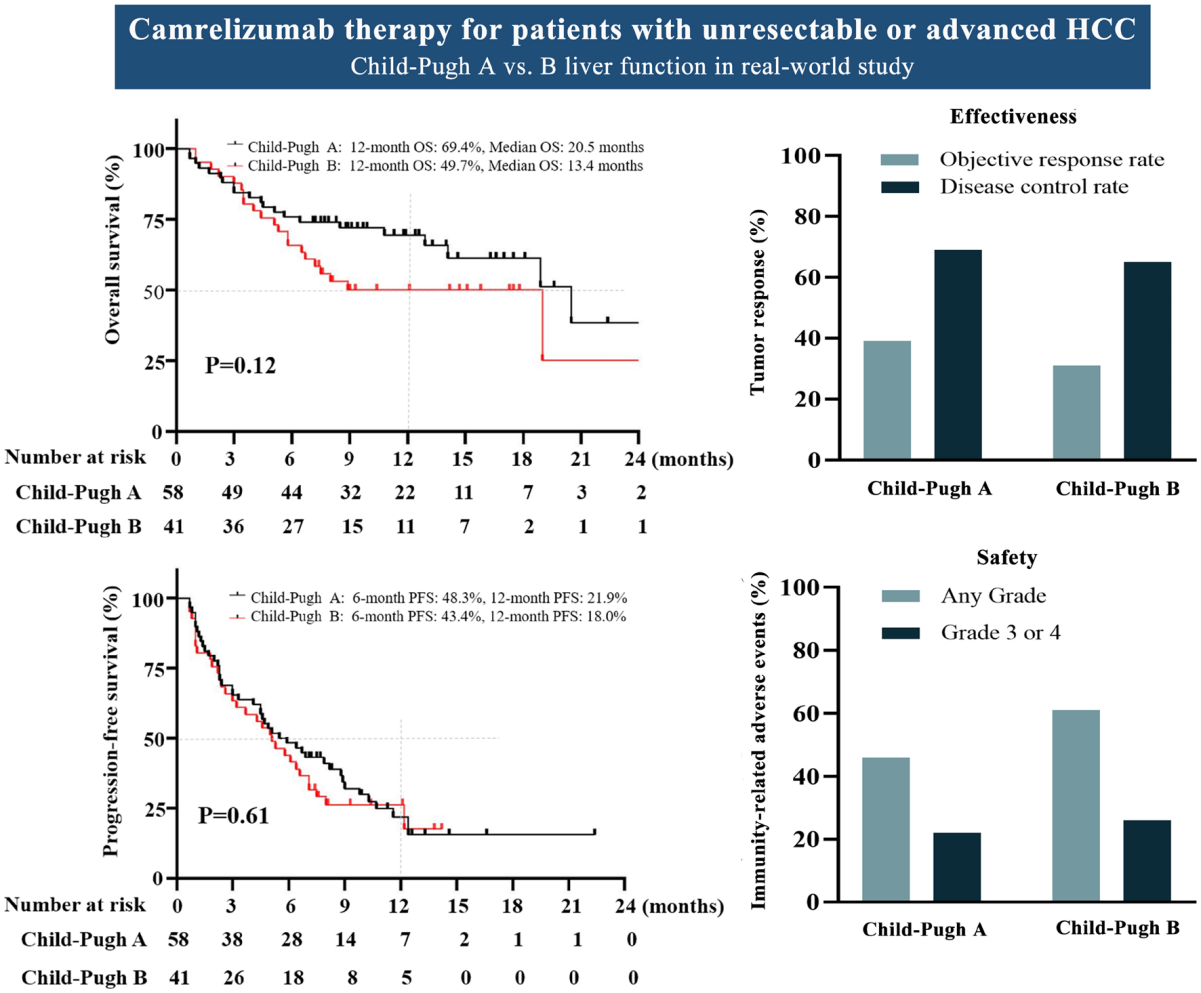

**Supplementary Information:**

The online version contains supplementary material available at 10.1007/s00262-023-03404-8.

## Introduction

Hepatocellular carcinoma (HCC) is the most common primary liver cancer and the fourth most common cause of cancer-related death worldwide [1, 2]. In China, HCC is the first and second most common cause of cancer-related death in males and females younger than 65 years of age, respectively [3, 4]. Patients with advanced HCC are generally not candidates for curative surgical treatment or even local treatment [5], making novel systemic therapies one of the main treatment options for this population.

Recent therapeutic advances with programmed cell death protein 1 (PD-1)-targeted immunotherapy (e.g., pembrolizumab, nivolumab, and camrelizumab) have shown promising results in phase II and/or phase III studies of advanced HCC [6–10]. However, the vast majority patients enrolled in clinical trials have Child-Pugh A disease, whereas many real-world patients with advanced HCC also have poor hepatic function with more advanced Child-Pugh class. Therefore, real-world effectiveness and tolerability data with PD-1 immunotherapy for patients with advanced HCC seen in routine practice are needed. An international multicenter real-world cohort study of 33 Child-Pugh B/C patients who received treatment with nivolumab or pembrolizumab reported comparable effectiveness and tolerability to Child-Pugh A patients [11]. Subgroup analysis of CheckMate 040 cohort 5 including 49 nivolumab-treated patients with a Child-Pugh score of 7–8 also showed similar efficacy and safety compared to Child-Pugh A patients [12]. However, no study has exclusively evaluated the effectiveness and tolerability of the PD-1 immunotherapy camrelizumab combined with molecular targeted therapy such as sorafenib, lenvatinib, and apatinib in patients with advanced HCC with Child-Pugh B liver function in the real world.

Therefore, we evaluated the effectiveness and tolerability of camrelizumab combined with molecular targeted therapy for unresectable or advanced HCC, focusing on patients with Child-Pugh B liver function in a multicenter retrospective real-world cohort study from China.

## Materials and methods

### Study subjects

We included adult patients (≥ 18 years of age) with unresectable or advanced HCC [defined as Barcelona Clinic Liver Cancer (BCLC) stage B/C or BCLC A, and had inadequate liver function to tolerate surgery including recurrent tumor after prior surgical resection] and Child-Pugh A or B who received at least one cycle of camrelizumab with or without combined molecular targeted therapy (lenvatinib, apatinib, sorafenib, regorafenib, or anlotinib) as part of their routine care at six medical centers in China between January 10, 2019, and March 31, 2021. Patients were followed up until death, loss to follow-up, or October 31, 2021, whichever came first. HCC was defined either by histology or radiology criteria [13]. Eligible patients had active HCC and tumor response was evaluated using the modified Response Evaluation Criteria in Solid Tumors (mRECIST) v1.1. Other inclusion criteria were Eastern Cooperative Oncology Group (ECOG) performance status ≤ 2, a predicted life expectancy of greater than 12 weeks, and Child-Pugh A or B liver function. The exclusion criteria were prior treatment with other immunotherapeutic agents; severe organ system complications such as severe cardiovascular diseases, chronic kidney disease, chronic obstructive pulmonary disease or asthma, brain or leptomeningeal metastasis or uncontrolled non-liver comorbidities; pregnant or breast feeding; presence of non-HCC malignant liver tumors; Child Pugh C; or incomplete imaging or Child-Pugh data.

### Treatment procedure

Eight-four patients received camrelizumab administered intravenously at a fixed dose of 200 mg every 3 weeks combined with targeted therapy including lenvatinib, apatinib, sorafenib, regorafenib, or anlotinib everyday. Fifteen patients received camrelizumab monotherapy. Dose delays were made based on tolerability and toxicity. Treatment was continued until disease progression, presence of unacceptable toxicity, whichever occurred first. Patients who had radiological disease progression were permitted to continue camrelizumab if the clinician determined that they would benefit from and could tolerate continued treatment.

### Assessments

Contrast-enhanced computed tomography or magnetic resonance imaging was performed at baseline, 6–12 weeks after treatment initiation, and about every 3 months thereafter. Tumor response was assessed according to mRECIST v1.1 [14]. Primary endpoints were tolerability and 12-month OS rate. The secondary endpoints were progression-free survival (PFS) and objective response rate (ORR) classified as complete response (CR) and partial response (PR). Those with CR, PR, or stable disease (those without CR or PR but without increase in tumor burden by 20% or more for at least 4 weeks) were considered to have reasonable disease control. PFS was defined as the time from treatment allocation to the first documented disease progression or death from any cause, whichever occurred first. OS was defined as the time from the first dose of study medication to death from any cause. ORR and disease control rate (DCR) were assessed in participants who underwent at least two efficacy evaluations after the first dose of study medication. Tolerability was recorded at every visit and graded according to the National Cancer Institute Common Terminology Criteria for Adverse Events v4.0.

### Statistical analyses

Data on baseline characteristics, tumor response, and side effects were summarized using descriptive statistics. The chi-square test or Fisher’s exact test was used to compare nominal data. Survival analyses were performed using the Kaplan–Meier method, and differences in the survival curves were analyzed with the log-rank test. Univariate and multivariate analyses were performed to determine the prognostic factors for OS and tumor response. Hazard ratios (HRs) and confidence intervals (CI) were also calculated. All variables with *P* < 0.10 in univariate analyses were included in the Cox proportional hazards model for multivariate analyses. To assess the association between primary or second endpoints and baseline variables, prespecified subgroup analyses were done based on the following factors: macrovascular invasion (MVI) (yes vs. no), alpha-fetoprotein (AFP) level (< 400 ng/mL vs. ≥ 400 ng/mL), extrahepatic metastasis (yes vs. no), BCLC stage (A/B vs. C), and Child-Pugh class (A vs. B, A/B7 vs. B8-9, A vs. B7 vs. B8-9). Two-tailed *P* < 0.05 was considered statistically significant. All data analyses were performed using SPSS 25.0 software (BM Corp., Armonk, NY, USA) and GraphPad Prism (version 8.0; GraphPad Software, San Diego, CA, USA).

## Results

### Patients’ baseline clinical characteristics

**Fig. 1:**
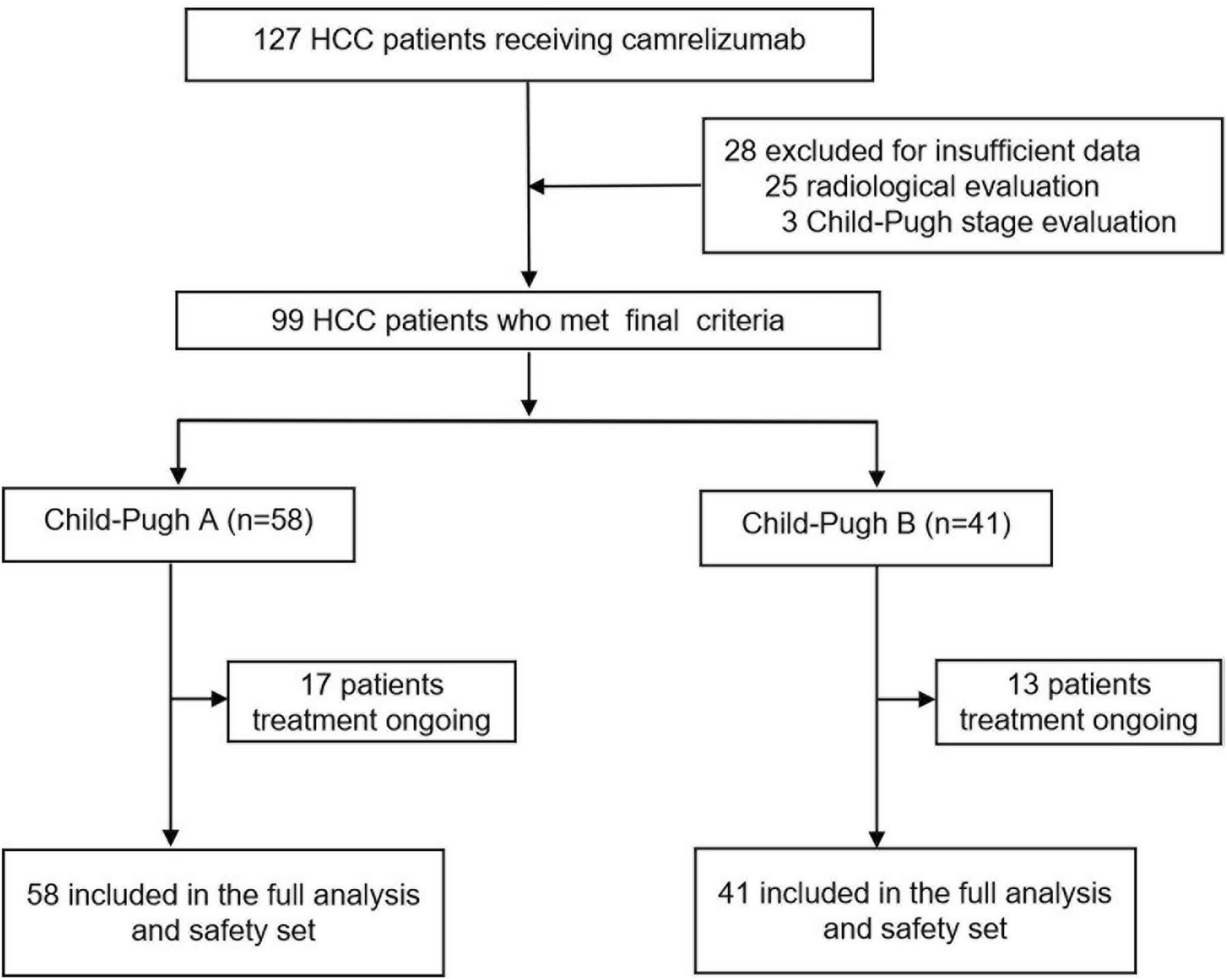
Patient flowchart

Between January 10, 2019, and March 31, 2021, 127 patients with HCC received camrelizumab with or without molecular targeted therapy; 99 patients were included in our study, including 41 Child-Pugh B and 58 Child-Pugh A patients (Fig. 1). The main baseline characteristics are shown in Table 1. Among the enrolled patients, 17 (17/99, 17.2%) received PD-1 inhibitors at the initial diagnosis of HCC and 82 patients had at least one previous HCC treatment including transarterial chemoembolization (TACE), radiofrequency ablation (RFA), surgical resection, or radiotherapy. Eighty four patients (84.8%) received tyrosine kinase inhibitors (TKIs) therapy for median 8.9 months (interquartile range 5.3–14.0 months), at the initial stage of immunotherapy including 48 (82.8%) and 36 (87.8%) Child-Pugh A and B patients, respectively. In all, 82 patients (82.8%) had advanced stage HCC, and 48 of 58 (82.8%) and 34 of 41(82.9%) were Child-Pugh stage A and B, respectively. The majority of patients (79.8%) had hepatitis B virus (HBV), and 43 patients (43.4%) had an AFP level ≥ 400 ng/mL. A total of 40 patients (40.4%) developed MVI, and 40 patients (40.4%) had extrahepatic metastasis including pulmonary metastasis (*n* = 21), osseous metastasis (*n* = 11), lymphatic metastasis (*n* = 13), and adrenal metastases (*n* = 3). The median duration of follow-up was 12.1 months (95% CI 9.9–14.0). In all, 42 patients (42.4%) died during follow-up including 21 Child-Pugh A (36.2%) and 21 Child-Pugh B (51.2%). The median number of cycles of camrelizumab treatment was six (95% CI 5–7). At data cutoff, 17 (29.3%) and 13 (31.7%) patients were still being treated with camrelizumab with or without TKIs.

**Table 1:** Baseline characteristics of patients with camrelizumab therapy

Characteristics	Overall (*n* = 99)	Child-Pugh A (*n* = 58)	Child-Pugh B (*n* = 41)	*p* value
*Sex*				0.289
Male	82 (82.8)	50 (86.2)	32 (78.0)	
Female	17 (17.2)	8 (13.8)	9(22.0)	
*Liver disease etiology*				0.167
HBV	79 (79.8)	49 (84.5)	30 (73.2)	
HCV	7 (7.1)	2(3.4)	5 (12.2)	
Non-HBV/HCV	13 (13.1)	7 (12.1)	6(14.6)	
*AFP (ng/mL)*				0.937
≥ 400	43 (43.4)	25(43.1)	18 (43.9)	
400	56 (56.6)	33 (56.9)	23 (56.1)	
*ALBI class*				0.000
1	18 (18.2)	17 (29.3)	1 (2.4)	
2	74 (74.7)	41(70.7)	33 (80.5)	
3	7 (7.1)	0 (0)	7 (17.1)	
*BCLC stage*				0.936
A	17 (17.2)	10 (17.2)	7 (17.1)	
B	21 (21.2)	13 (22.4)	8 (19.5)	
C	61 (61.6)	35 (60.3)	26 (63.4)	
*Macrovascular invasion*				0.456
No	59 (59.6)	36 (62.1)	23 (56.1)	
Yes	40 (40.4)	22 (37.9)	18 (43.9)	
*Extrahepatic metastases*				0.857
No	59 (59.6)	35 (60.3)	24 (58.5)	
Yes	40 (40.4)	23 (39.7)	17 (41.5)	
Pulmonary metastasis	21 (52.5)	15 (25.9)	6(14.6)	
Osseous metastasis	11 (27.5)	5(8.6)	6 (14.6)	
Lymphatic metastasis	13 (32.5)	9 (15.5)	4 (9.8)	
Adrenal metastases	3 (7.5)	2(3.4)	1 (2.4)	
Others	3 (7.5)	1 (1.7)	2(4.9)	
Previous HCC treatments				0.000
None	17 (17.2)	3(5.2)	14 (34.1)	
TACE	73 (73.7)	50 (86.2)	23 (56.1)	
Surgical resection	21 (21.2)	18 (31.0)	3(7.3)	
RFA	35 (35.4)	22 (37.9)	13 (31.7)	
Radiotherapy	5 (5.1)	2(3.4)	3(7.3)	
Surgical resection + TACE	16 (16.2)	14 (24.1)	2(4.9)	
RFA + TACE	30 (30.3)	21 (36.2)	9 (22.0)	
≥ 2 treatments	39 (39.4)	28 (43.3)	11 (26.8)	
*Molecular targeted therapy*^a^				0.367
No	15 (15.2)	10 (17.2)	5 (12.2)	
Yes	84 (84.8)	48 (82.8)	36 (87.8)	
Lenvatinib	38 (38.4)	23 (39.7)	15 (36.6)	
Apatinib	26 (26.3)	13 (22.4)	13 (31.7)	
Sorafenib	26 (26.3)	17 (29.3)	9 (22.0)	
Regorafenib	7 (7.1)	7 (12.1)	0	
Anlotinib	3 (3.0)	2(3.4)	1 (2.4)	
*ECOG, physical status score*				0.185
0	22 (22.2)	15 (25.9)	7(17.1)	
1	58 (58.6)	35 (60.3)	23 (56.1)	
2	19 (19.2)	8 (13.8)	11 (26.8)	
Age in years	58 (30–83)	56.5 (35–80)	58 (30–83)	0.224
WBC as 10^9^/L	5.05 ± 2.66	5.2 ± 3.17	4.95 ± 2.25	0.045
HB in g/L	125.28 ± 21.91	131.74 ± 19.66	115.9 ± 22.12	0.006
PLT as 10^9^/L	108 (30–437)	113.5(34–437)	98 (30–314)	0.017
PT in s	12.74 ± 1.56	12.31 ± 1.36	13.35 ± 1.65	0.005
ALT in IU/L	40 (10.3–261)	34.2(10.3–146.6)	41.8(15–461)	0.017
AST in IU/L	54 (17.1–522)	50.9(17.1–522)	65 (23–188.8)	0.019
ALB in g/L	35 (23.5–48.8)	36.8 (29.8–48.8)	31.1 (23.5- 41.3)	0.006
TBILin mmol/L	17.8 (4.8–108.2)	16.15 (4.8–50.8)	31.1 (10–108.2)	0.012

Data are presented as *n* (%), median (range), or mean (± SD)

*AFP* alpha-fetoprotein, *ALB* albumin, *ALBI* albumin–bilirubin, *ALT* alanine transaminase, *AST* aspartate transaminase, *BCLC* Barcelona Clinic Liver Cancer, *ECOG* Eastern Cooperative Oncology Group, *HB* hemoglobin, *HBV* hepatitis B virus, *HCC* hepatocellular carcinoma, *HCV* hepatitis C virus, *PLT* platelet, *PT* prothrombin time, *RFA* radiofrequency ablation, *TACE* transarterial chemoembolization, *TBIL* total bilirubin, and *WBC* white blood cell

^a^Of the 84 patients who received camrelizumab combined with molecular targeted therapy, 69 patients are molecular targeted therapy naive, and 15 patients had sorafenib, lenvatinib or anlotinib treatment failure

### Effectiveness in the overall cohort and by Child-Pugh stage

**Fig. 2:**
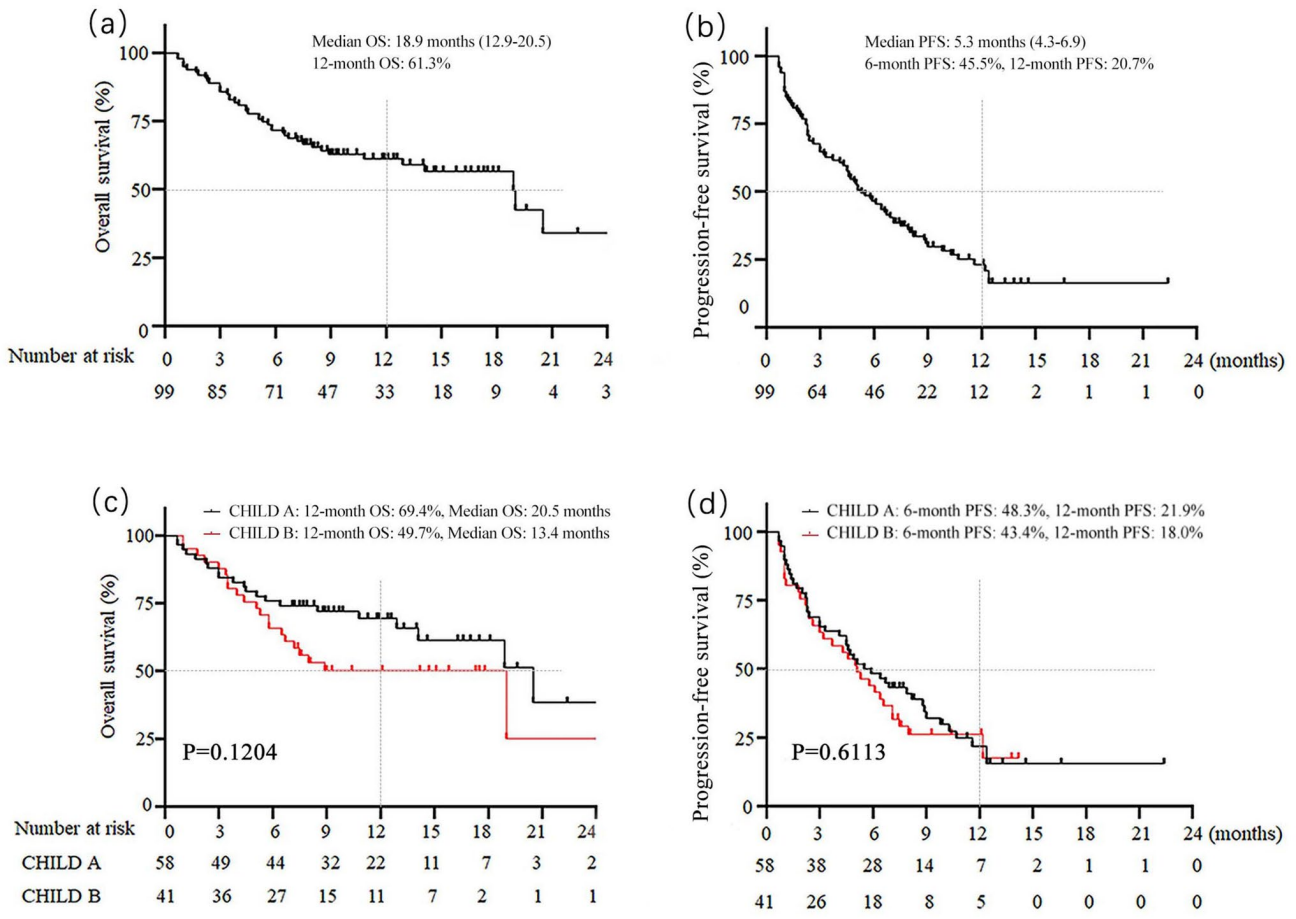
Overall survival and progression-free survival in the overall cohort (**a**, **b**) and subgroup by Child-Pugh stage (**c**, **d**)

The tumor response results are shown in Table 2. Seven patients had CR and twenty-nine (29.3%) had PR, resulting in an ORR of 36.4%. Thirty-one (31.3%) patients showed stable disease, and 32 (32.3%) subjects had progressive disease at the first radiological evaluation. The overall DCR was 67.7%. The ORR and DCR in Child-Pugh A and B were similar (Table 2). The median OS time was 18.9 months (95% CI 12.9–20.5) for the whole cohort (Fig. 2a), with no significant difference between groups (*p* = 0.12), although the median OS was shorter in Child-Pugh B patients (20.5 vs.13.4 months) (Fig. 2c). The 12-month OS rates were 61.3% for the whole cohort and 49.7% in the Child-Pugh B group, which were comparable to Child-Pugh A patients (Fig. 2c, Table 2). The median PFS was 5.3 months (95% CI 4.3–6.9) for the whole group (Fig. 2b), and 5.5 (95% CI 4.1–8.8) and 5.1 (95% CI 2.6–7.1) months for Child-Pugh A and B patients, respectively (Fig. 2d, Table 2). The 6- and 12-month PFS rates were 45.5% and 20.7%, respectively, for the whole cohort and were comparable in both groups (Fig. 2d).

**Table 2:** Assessable radiological response and survival

Variable	Overall response mRECIST		
	Overall (*n* = 99)	Child-Pugh A (*n* = 58)	Child-Pugh B (*n* = 41)
CR	7 (7.1)	3 (5.2)	4 (9.8)
PR	29 (29.3)	20 (34.5)	9 (22.0)
SD	31 (31.3)	17 (29.3)	14 (34.1)
PD	32 (32.3)	18 (31.0)	14 (34.1)
ORR	36 (36.4)	23 (39.7)	13 (31.7)
DCR	67 (67.7)	40 (69.0)	24 (65.9)
PFS in months	5.3 (4.3–6.9)	5.5 (4.1–8.8)	5.1 (2.6–7.1)
6	45.5%	48.3%	43.4%
12	20.7%	21.9%	18.0%
OS in months	18.9 (12.9–20.5)	20.5 (14.1–20.5)	13.4 (5.8–19.0)
12	61.3%	69.4%	49.7%

Data are presented as n (%) or median (95% CI)

*CI* confidence interval, *CR* complete response, *DCR* disease control rate (CR + PR + stable disease), *ORR* objective response rate (CR + PR), *OS* overall survival, *PD* progressive disease, *PFS* progression-free survival, *PR* partial response, and *SD* stable disease

To further clarify the effect of liver function on patient survival, we evaluated it in subgroups (Supplementary Fig. 1). There was no difference in median OS in Child-Pugh A/B7 and Child-Pugh B8-9 patients (*p* = 0.374), as well as in Child-Pugh A, Child-Pugh B7, and Child-Pugh B8-9 patients (*p* = 0.298). Considering the poor prognosis in BCLC stage C patients, we stratified the effects of different liver functions on the survival of BCLC stage C patients. There was only a lower trend in survival in Child-Pugh B patients, and there was no difference in OS in Child-Pugh A/B7 versus B8-9 patients (*p* = 0.846), and Child-Pugh A versus B7 versus B8-9 patients (*p* = 0.223).

**Fig. 3:**
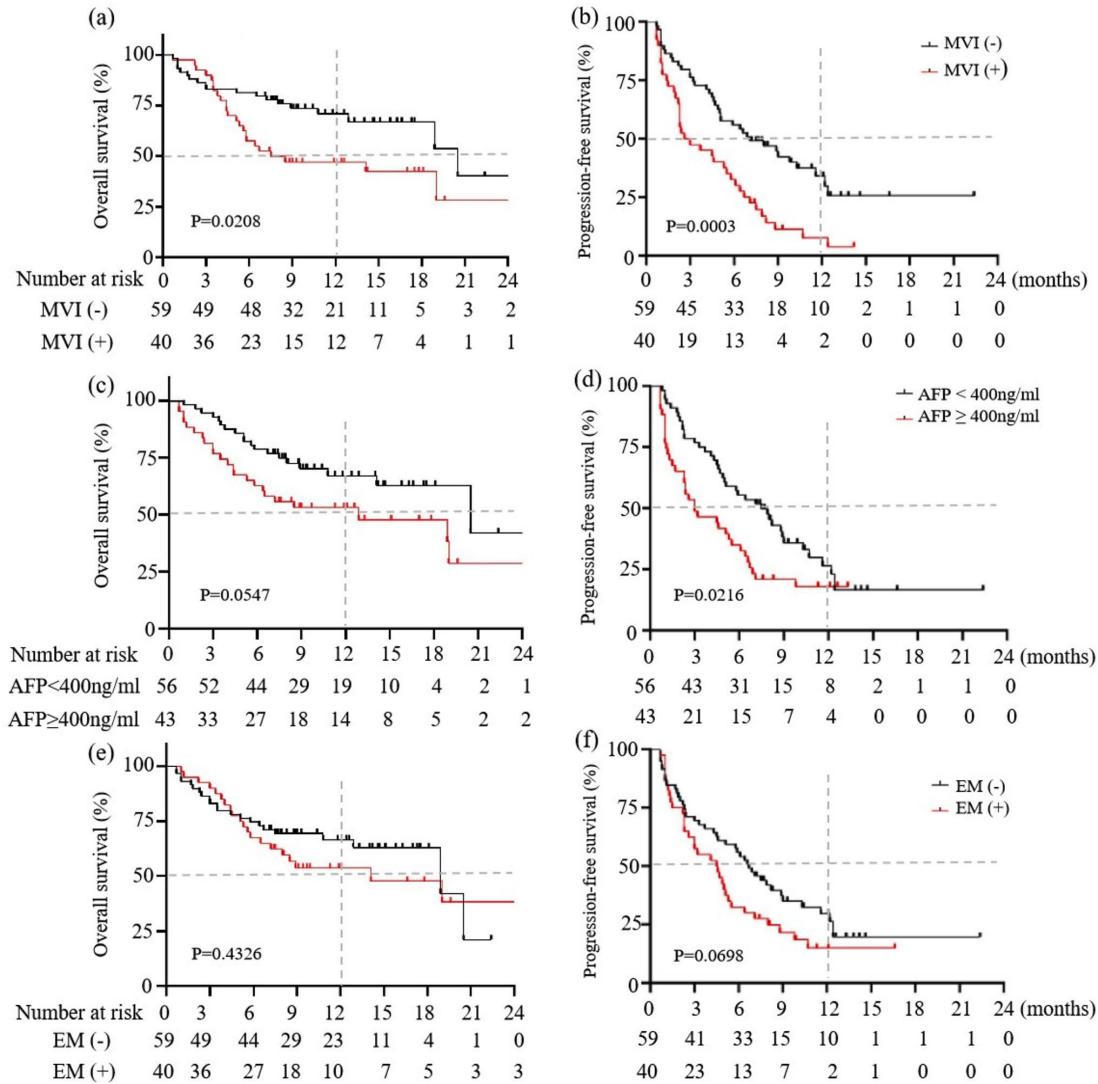
Overall survival and progression-free survival by subgroups in (**a**, **b**) with and without macrovascular invasion; (**c**, **d**) alpha-fetoprotein (AFP) < 400 ng/mL versus AFP ≥ 400 ng/mL; (**e**, **f**) with and without extrahepatic metastasis. *MVI* macrovascular infiltration, *EM* extrahepatic metastasis

The median OS was 7.5 months (95% CI 5.3–19.0) in patients with MVI, which was lower than the 20.5 months in patients without MVI (95% CI 18.9–20.5; *p* = 0.021) (Fig. 3a). The median PFS was 2.6 (95% CI 2.2–5.5) and 7.1 (95% CI 4.9–11.6) months for patients with or without MVI (*p* = 0.0003) (Fig. 3b). Patients with AFP ≥ 400 ng/mL showed a trend of poor survival compared to patients with AFP < 400 ng/mL (median OS: 12.9 vs. 14.1 months; *p* = 0.055) (Fig. 3c). The median PFS was 3.0 (95% CI 1.7–5.5) and 7.5 (95% CI 4.9–9.0) months for patients with AFP ≥ 400 ng/mL or < 400 ng/mL (*p* = 0.022) (Fig. 3d). However, there was no difference in median OS and PFS between patients with or without extrahepatic metastasis (both *p* > 0.05) (Fig. 3e, f).

There were no difference in OS or PFS between camrelizumab monotherapy and camrelizumab plus TKIs combination therapy groups (Supplementary Fig. 2) among the overall cohort. By Child-Pugh stage, among Child A patients (Supplementary Fig. 3a and b), there were also no significant differences in either OS or PFS between the ICI vs. the ICI + TKI groups. However, among Child B patients (Supplementary Fig. 3c and d), OS was higher in the ICI + TKI group compared with the ICI monotherapy group, whereas there was no significant difference between the two study groups for PFS. Of the 7 patients who had CR, 6 received ICI + TKIs combination therapy, and 1 patient received ICI monotherapy (Table S1).

### Prognostic factor analysis

Prognostic risk factors for 12-month OS and ORR were analyzed. In univariate analysis, BCLC stage C (*p* = 0.008), MVI (*p* = 0.01), and Child-Pugh B status (*p* = 0.049) were associated with failure to achieve 12-month OS; and age ≥ 60 (*p* = 0.031) and lack of MVI (*p* = 0.018) were associated with achieving ORR (Table S2). In multivariate analysis, MVI but not sex, age, HBV etiology, distant metastasis, lack of targeted therapy, Child-Pugh B status, or AFP > 400 ng/mL was associated with 12-month OS (HR 2.970, 95% CI 1.276–6.917; *p* = 0.012) and ORR (HR 2.906, 95% CI 1.18–7.16; *p* = 0.020) (Table S3).

### Tolerability in the overall cohort and by Child-Pugh stage

Fifty-two (52.5%) patients experienced at least one immune-related adverse event (irAE) (Table 3). The most common AEs were immune thrombocytopenia (*n* = 28, 28.3%), hepatotoxicity (*n* = 16, 16.2%), pruritus (*n* = 15, 15.2%), diarrhea (*n* = 15, 15.2%), hypothyroidism (*n* = 15, 15.2%), maculopapule (*n* = 13, 13.1%), cutaneous capillary endothelial proliferation (CCEP) (*n* = 11, 11.1%), hyperglycemia (*n* = 7, 7.1%), and cardiotoxicity (*n* = 7, 7.1%). About one-quarter of patients (24.2%) developed AEs of higher grade (grade ≥ 3). Hepatotoxicity (*n* = 13, 13.1%) and immune thrombocytopenia (*n* = 9, 9.1%) were the most common severe AEs, followed by hypophysitis (*n* = 4, 4.0%) and primary adrenal hypofunction (*n* = 4, 4.0%). One Child-Pugh B patient experienced severe immune thrombocytopenia, hepatotoxicity, cardiotoxicity, primary adrenal hypofunction, and death from multiple organ failure, despite receiving 80 mg methylprednisolone and 5 mg/kg immunoglobulin based on body weight intravenously for 5 days, as well as platelet infusion; the death was determined to be potentially treatment-related. Severe AEs led to discontinuation of camrelizumab treatment in all 24 patients; camrelizumab was re-initiated in 6 of them based on the clinician determining that the patients would benefit from continued treatment. In terms of tolerability, any grade and grade 3/4 irAEs occurred in 61% and 26.8% patients with Child-Pugh B, respectively, and there was no difference in patients who developed any grade or grade 3/4 between Child-Pugh stage B and A status. AEs according to Child-Pugh stage are shown in Table 3.

**Table 3:** Immune-related adverse events during camrelizumab treatment

Event (*n* = 99)	Any grade, *n* (%)	Grade 3 or 4, *n* (%)		Child-Pugh A (*n* = 58)		Child-Pugh B (*n* = 41)		
				Any grade, *n* (%)	Grade 3 or 4, *n* (%)		Any grade, *n* (%)	Grade 3 or 4, *n* (%)
Any irAEs	52 ( 52.5)	24 (24.2)		27 ( 46.6)	13 ( 22.4)		25 (61.0)	11 ( 26.8)
Hepatotoxicity	16 (16.2)	13 (13.1)		8 (13.8)	7 (12.1)		8 (19.5)	6 (14.6)
Immune thrombocytopenia	28 (28.3)	9 (9.1)		14 (24.1)	3 (5.2)		14 (34.1)	6 (14.6)
Diarrhea	15 (15.2)	1 (1.0)		9 (15.5)	0 (0.0)		6 (14.6)	1 (2.4)
Hypothyroidism	15 (15.2)	0 (0.0)		8 (13.8)	0 (0.0)		7 (17.1)	0 (0.0)
Pruritus	15 (15.2)	0 (0.0)		8 (13.8)	0 (0.0)		7 (17.1)	0 (0.0)
Maculopapule	13 (13.1)	0 (0.0)		7 (12.1)	0 (0.0)		6 (14.6)	0 (0.0)
CCEP	11 (11.1)	0 (0.0)		7 (12.1)	0 (0.0)		4 (9.8)	0 (0.0)
Hyperglycemia	7 (7.1)	0 (0.0)		6 (10.3)	0 (0.0)		1 (2.4)	0 (0.0)
Cardiotoxicity	7 (7.1)	1 (1.0)		2 (3.4)	0 (0.0)		5 (12.2)	1 (2.4)
Hypophysitis	4 (4.0)	4 (4.0)		3 (5.2)	3 (5.2)		1 (2.4)	1 (2.4)
Primary adrenal hypofunction	4 (4.0)	4 (4.0)		3 (5.2)	3 (5.2)		1 (2.4)	1 (2.4)
Hyperthyroidism	2 (2.0)	1 (1.0)		0 (0.0)	0 (0.0)		2 (4.9)	1 (2.4)
Nephrotoxicity	3 (3.0)	0 (0.0)		1 (1.7)	0 (0.0)		2 (4.9)	0 (0.0)
Pulmonary toxicity	1 (1.0)		1 (1.0)	1 (1.7)	1 (1.7)		0 (0.0)	0 (0.0)

*CCEP* cutaneous capillary endothelial proliferation, *irAEs* immune-related adverse events

## Discussion

Our study demonstrated that camrelizumab combined with molecular targeted therapy showed favorable effectiveness and tolerability with manageable toxicities in a real-world Chinese cohort of patients with unresectable or advanced stage HCC. Effectiveness and tolerability were comparable between Child-Pugh A and B patients, even though the median OS was shorter in Child-Pugh B patients (20.5 vs.13.4 months). For Child-Pugh B patients, the ORR and DCR were 31.7% and 65.9%, respectively, and the median PFS was 5.1 months, which were comparable to Child-Pugh A patients. MVI at initial admission was independently associated with 12-month OS and ORR in patients who received anti-PD-1 combined therapy. The OS of patients with disease stabilization was significantly longer than that of patients with progressive disease (Supplementary Fig. 4).

Studies have assessed the efficacy and safety of nivolumab or pembrolizumab in aHCC patients with Child-Pugh B liver function [11, 12]. In the study by Scheiner et al. [11], of the 33 patients with Child-Pugh stage B/C disease, the ORR and DCR were 14% and 46%, respectively, and the median PFS and OS were 4.6 and 8.6 months, respectively [11]. In a phase I/II study of nivolumab in patients with advanced HCC, Kudo et al. [12]. reported 49 sorafenib-naive or sorafenib-treated Child-Pugh B patients with cirrhosis (76% and 24% patients had a Child-Pugh score of B7 and B8, respectively); the ORR and DCR were 12% and 55%, respectively, and the median OS was 7.6 months [12]. All of these indicators including ORR, DCR, and OS were comparable to Child-Pugh A patients in these studies [11, 12]. However, both studies included a small number of patients with HBV infection.

Treatment with camrelizumab every 2 or 3 weeks in a large Chinese cohort of previously treated Child-Pugh A patients with aHCC, including 83% patients with HBV infection, led to an ORR of 14.7% and median OS of 13.8 months (11.5–16.6) [10]. Furthermore, camrelizumab in combination with apatinib in treatment-naive and pretreated patients with aHCC led to a substantial number of objective responses (22.5–34.3%), prolonged median PFS (5.5–5.7 months), and a 12-month OS rate of 68.2–74.7% [9]. Anti-vascular endothelial growth factor-targeted therapies can induce hypoxia and promote an immunosuppressive tumor microenvironment by upregulating immune checkpoint molecules [15, 16]; thus, the combination of anti-angiogenic agents with immunotherapy is particularly attractive. Indeed, substantial improvement in tumor response rate also was reported in two Chinese studies that employed anti-PD-1 plus targeted therapy with lenvatinib or anlotinib [17, 18]. However, those studies only included Child-Pugh A patients or a few Child-Pugh B7 patients; thus, the tolerability and effectiveness of camrelizumab combined with targeted therapy for advanced HCC in patients with severe liver insufficiency remain a substantial unmet clinical need, especially in this population that has a poor prognosis. The high tumor response rate in our study may be because the majority of patients (84.8%) received TKIs combination therapy.

HCC patients with HBV infection are thought to have poorer prognoses than patients with HCV infection [19, 20]. The effect of etiology on efficacy in HCC patients receiving anti-PD-1 treatment remains unclear, despite the fact that the efficacy of nivolumab and pembrolizumab was not affected by HBV or HCV infection in sorafenib-treated patients in phase 1/2 trials [8, 21, 22]. The majority of patients in our study had HBV infection and a good tumor response, suggesting that HCC etiology does not have a significant impact on camrelizumab survival outcomes. These data provide important new information on advanced HCC patients with Child-Pugh B status, who are often excluded from receiving immunotherapy or targeted therapy in clinical practice. To the best of our knowledge, this is the first study to report anti-PD-1 plus targeted therapy for unresectable or advanced HCC patients with Child-Pugh B liver function in real-world clinical practice, which will provide an alternative treatment option for this population, especially in China and Southeast Asia, where HBV is highly prevalent.

Tolerability was important concerns in our study. Overall, camrelizumab therapy for advanced HCC in Child-Pugh B patients led to 61% immune-related any grade AEs and 26.8% AEs of grade 3 or 4, consistent with those in Child-Pugh A patients, and no new safety concerns were observed. Severe AEs led to treatment interruption in 11 Child-Pugh A patients (19.0%) and 7 Child-Pugh B patients (17.1%), including one treatment-related death in the latter group. The spectrum of AEs was similar to that of other PD-1 immune checkpoint inhibitors, except for the occurrence of reactive CCEP. In a recent study, 145 of the 217 patients (66.8%) treated with camrelizumab monotherapy experienced grade 1/2 CCEP, which was associated with a higher tumor response [10, 23]. The risk of CCEP significantly decreased to 14.3–29.5% when camrelizumab was combined with lenvatinib or apatinib [9, 17]. Eleven patients (11.1%, most received camrelizumab monotherapy) experienced CCEP (grade 1 or 2) mainly on the face, hand, trunk, and skin. The low incidence of CCEP may be related to targeted therapy against capillary endothelial proliferation. The CCEP was clinically controllable and self-limiting, and usually present in the first 4 weeks and alleviated at 10–12 weeks; the underlying mechanisms need to be further clarified. No patients discontinued camrelizumab due to CCEP.

Another important safety concern was immunotherapy-related liver injury, which has been reported in up to 20% of patients depending on the agent(s) used and underlying factors [24]. In particular, Child-Pugh B patients are more vulnerable to liver function impairment. Six Child-Pugh B patients (14.6%) experienced grade 3 or 4 hepatotoxicity, suggesting that closely monitoring liver function is mandatory in this population. The underlying mechanisms of hepatotoxicity are not fully understood. Blocking the PD-1/PD-L1 axis may lead to the destruction of hepatocytes due to HBV reactivation [25]; however, it remains unknown whether HBV reactivation contributes to hepatotoxicity, as we had insufficient data to assess the relationship between HBV viral load and dysfunction, although all HBsAg-positive patients received nucleoside (acid) drugs therapy.

This study had some limitations. First, the study design was retrospective and included a relatively small sample size and short-term follow-up. Second, the majority of patients were treated with very different modalities including surgery, targeted therapies, RFA and TACE, and in different time intervals, thus negatively impacting comparisons of the primary endpoints between Child-Pugh B and A groups. Third, we did not establish uniform guidelines to assess irAEs. Moreover, the mild AEs may not have been recorded, which could have led to an underestimation of AE frequency in our cohort. Fourth, although all HBV patients had baseline HBV DNA load, we had insufficient data to evaluate HBV reactivation, in particular, our cohort contained a high proportion of patients with HBV etiology. Finally, hyperprogressive disease has been reported in 8–12.7% advance HCC patients received anti-PD-1 therapy [11, 26], but there were insufficient data to evaluate HCC hyperprogression in this study, although seven patients had rapid disease progression after the first cycle of anti-PD-1 therapy. Despite these limitations, we were still able to generate strong conclusions after careful analysis of the data.

In conclusion, camrelizumab combined with molecular targeted therapy showed clinical activity and favorable safety with manageable toxicities in Chinese patients with advanced HCC, regardless of Child-Pugh A/B liver function, suggesting that it could be suitable for this population, even with a high proportion of patients with HBV infection. Significantly improved survival in Child-Pugh B patients who achieved disease stabilization support that immunotherapy should be attempted in this population. The tumor responses should be assessed early and AEs should be closely monitored to help confirm individuals who can benefit from immunotherapy. MVI is associated with a suboptimal immunotherapy response and poor prognosis.

### Supplementary Information

Below is the link to the electronic supplementary material.

Supplementary file1 (TIF 841 kb)

Supplementary file2 (TIF 747 kb)

Supplementary file3 (TIF 1221 kb)

Supplementary file4 (TIF 539 kb)

Supplementary file5 (DOCX 19 kb)

Supplementary file6 (DOCX 13 kb)

## Abbreviations

AEs: Adverse events
AFP: Alpha-fetoprotein
aHCC: Advanced hepatocellular carcinoma
BCLC stage: Barcelona clinic liver cancer stage
CCEP: Cutaneous capillary endothelial proliferation
CI: Confidence intervals
DCR: Disease control rate
ECOG: Eastern cooperative oncology group
HBV: Hepatitis B virus
HCC: Hepatocellular carcinoma
HCV: Hepatitis C virus
HRs: Hazard ratios
irAE: Immune-related adverse event
mRECIST: Modified response evaluation criteria in solid tumors
MVI: Macrovascular infiltration
ORR: Objective response rate
OS: Overall survival
PD-1: Programmed cell death protein 1
PFS: Progression-free survival
RFA: Radiofrequency ablation
TACE: Transarterial chemoembolization

## Declarations

### Availability of data and material

All data and materials of this study are available from the corresponding author upon request.
