# Supplementary figures and images for: Effectiveness and tolerability of camrelizumab combined with molecular targeted therapy for patients with unresectable or advanced HCC
Source: Cancer Immunol Immunother. 2023 Feb 25;72(7):2137–49. doi: 10.1007/s00262-023-03404-8 (PMC10264531; doi:10.1007/s00262-023-03404-8)

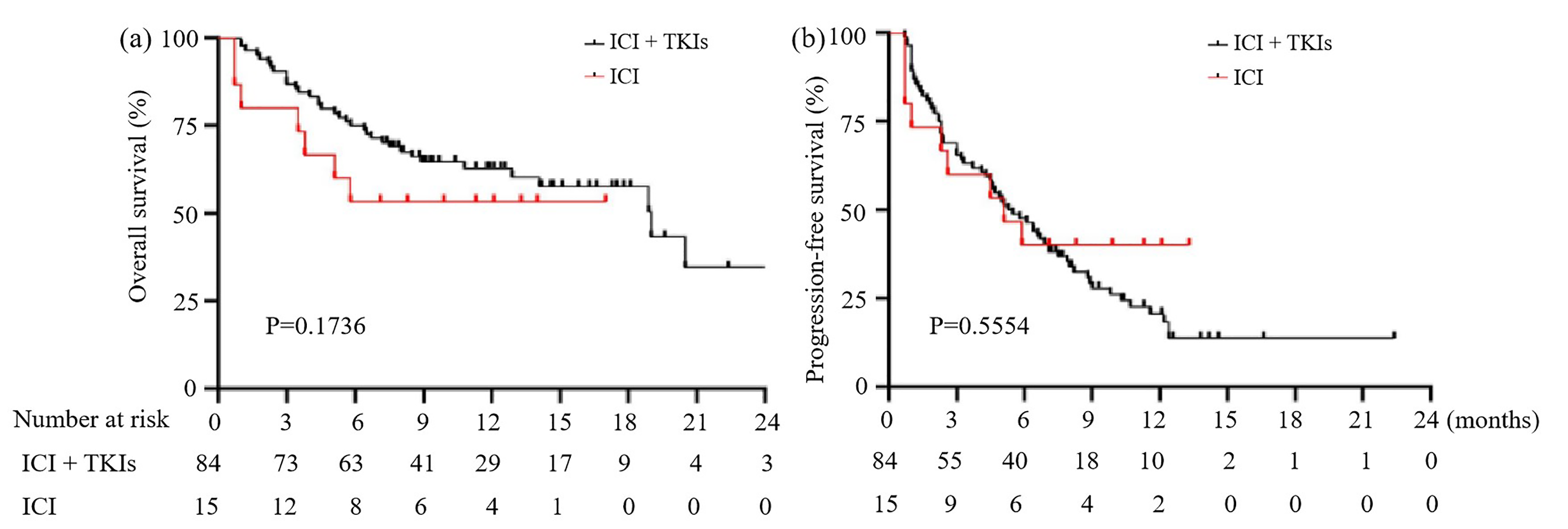

Supplement: Supplementary file 2 — Supplementary file2 (TIF 747 kb) [file 262_2023_3404_MOESM2_ESM.tif]

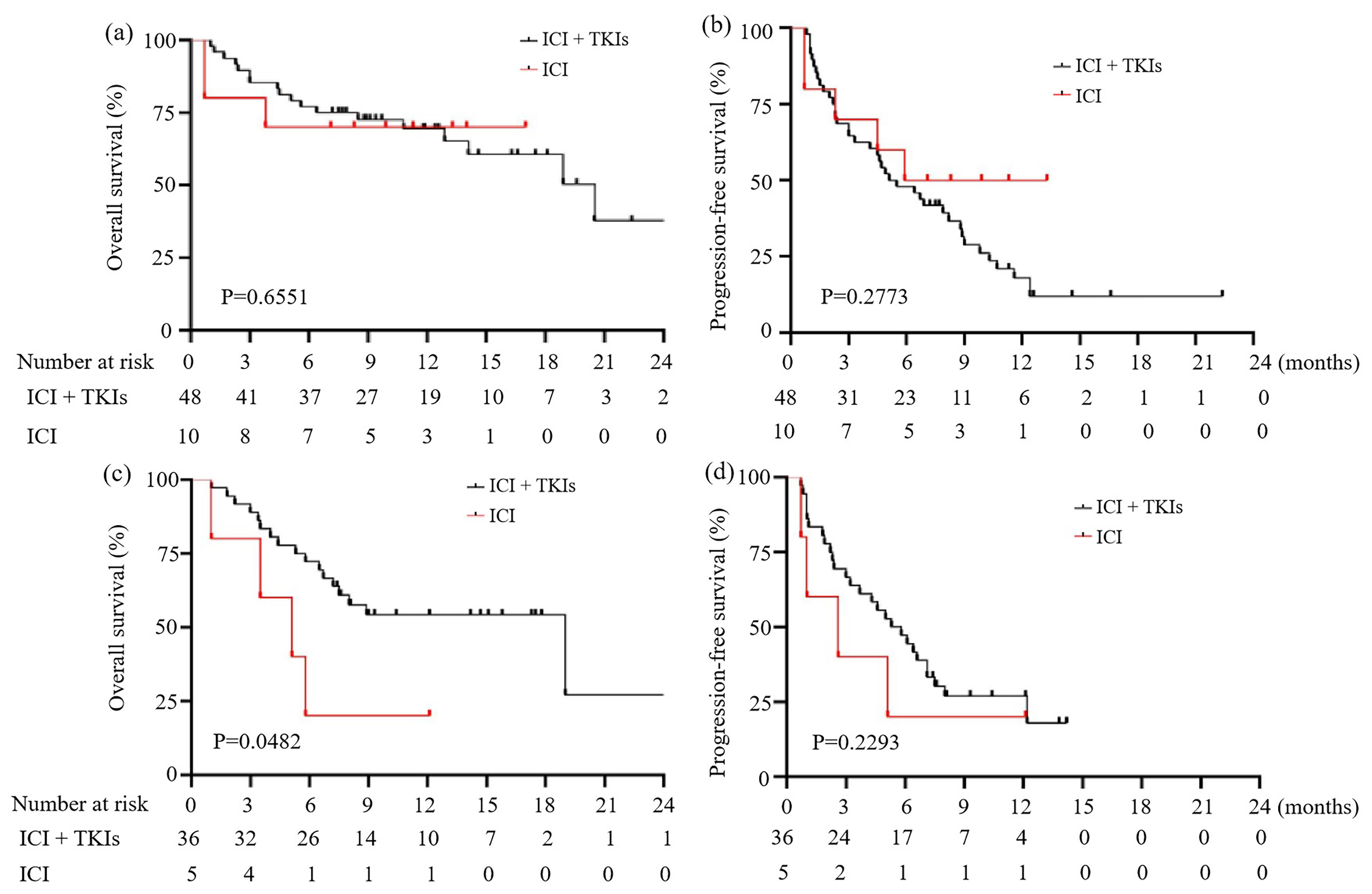

Supplement: Supplementary file 3 — Supplementary file3 (TIF 1221 kb) [file 262_2023_3404_MOESM3_ESM.tif]
