# Supplementary material for: Effectiveness and tolerability of camrelizumab combined with molecular targeted therapy for patients with unresectable or advanced HCC
Source: Cancer Immunol Immunother. 2023 Feb 25;72(7):2137–49. doi: 10.1007/s00262-023-03404-8 (PMC10264531; doi:10.1007/s00262-023-03404-8)
Supplement: Supplementary file 5 — Supplementary file5 (DOCX 19 kb) [file 262_2023_3404_MOESM5_ESM.docx]

**Supplementary Table 1.** The [therapeutic regimen](javascript:;)s in seven patients with complete response.

| **Complete response cases** | **Tyrosine kinase**  **inhibitors** | **Treatment prior to immunotherapy** |
| --- | --- | --- |
| Patient 1 | None | Transarterial chemoembolization,  Radiofrequency ablation |
| Patient 2 | Lenvatinib | Transarterial chemoembolization,  Radiofrequency ablation |
| Patient 3 | Sorafenib | Transarterial chemoembolization |
| Patient 4 | Lenvatinib | None |
| Patient 5 | Lenvatinib | Sorafenib, Transarterial chemoembolization,  Radiofrequency ablation, Radiotherapy |
| Patient 6 | Lenvatinib | Sorafenib, Transarterial chemoembolization |
| Patient 7 | Sorafenib | Transarterial chemoembolization,  Radiofrequency ablation, Radiotherapy |

**Supplementary Table 2.** Factors associated with 12-month OS and ORR in

univariate analysis.

| **Factors** | **12-month OS** | **P-value** | **ORR** | **P-value** |
| --- | --- | --- | --- | --- |
| **Sex** |  | 0.195 |  | 0.650 |
| male | 49 (59.8%) |  | 29 (35.4%) |  |
| female | 13 (76.5%) |  | 7 (41.2%) |  |
| **Age (years)** |  | 0.327 |  | 0.031 |
| <60 | 34 (58.6%) |  | 16 (27.6%) |  |
| ≥60 | 28 (68.3%) |  | 20 (48.8%) |  |
| **Etiology** |  | 0.806 |  | 0.705 |
| HBV | 49 (62.0%) |  | 28 (35.4%) |  |
| Non-HBV | 13 (65.0%) |  | 8 (40.0%) |  |
| **AFP (ng/mL)** |  | 0.100 |  | 0.490 |
| ≤400 | 39 (69.6%) |  | 22 (39.3%) |  |
| >400 | 23 (53.5%) |  | 14 (32.6%) |  |
| **BCLC staging** |  | 0.008 |  | 0.154 |
| A | 11 (64.7%) |  | 7 (41.2%) |  |
| B | 19 (90.5%) |  | 11 (52.4%) |  |
| **C** | 32 (52.5%) |  | 18 (29.5%) |  |
| **MVI** |  | 0.01 |  | 0.018 |
| No | 43 (72.9%) |  | 27 (45.8%) |  |
| Yes | 19 (47.5%) |  | 9 (22.5%) |  |
| **Extrahepatic metastases** | | 0.197 |  | 0.816 |
| No | 40 (67.8%) |  | 22 (37.3%) |  |
| Yes | 22 (55.0%) |  | 14 (35.0%) |  |
| **Targeted therapy** | | 0.565 |  | 0.642 |
| No | 9 (56.3%) |  | 5 (31.3%) |  |
| Yes | 53 (63.9%) |  | 31 (37.3%) |  |
| **Child-Pugh grading** | | 0.049 |  | 0.418 |
| A | 41 (70.7%) |  | 23 (39.7%) |  |
| B | 21 (51.2%) |  | 13 (31.7%) |  |
| **ALBI grading** | | 0.248 |  | 0.407 |
| 1 | 14 (77.8%) |  | 9 (50.0%) |  |
| 2 | 43 (58.1%) |  | 25 (33.8%) |  |
| 3 | 5 (71.4%) |  | 2 (28.6%) |  |

Note: ALBI: albumin-bilirubin; BCLC: Barcelona Clinic Liver Cancer; HBV: hepatitis B virus; MVI: macrovascular infiltration; ORR: objective response rate; OS: overall survival

**Supplementary Table 3.** Factors associated with 12-month OS and ORR in multivariate analysis.

|  | **Related factors** | **Regression coefficient** | **HR** | **95% CI** | **P-value** |
| --- | --- | --- | --- | --- | --- |
| **12-month OS** | MVI | 1.089 | 2.970 | 1.276-6.917 | 0.012 |
| **ORR** | MVI | 1.067 | 2.906 | 1.18-7.16 | 0.020 |

Note: MVI: macrovascular infiltration; ORR: objective response rate; OS: overall survival
