# Supplementary material for: Effectiveness and tolerability of camrelizumab combined with molecular targeted therapy for patients with unresectable or advanced HCC
Source: Cancer Immunol Immunother. 2023 Feb 25;72(7):2137–49. doi: 10.1007/s00262-023-03404-8 (PMC10264531; doi:10.1007/s00262-023-03404-8)
Supplement: Supplementary file 6 — Supplementary file6 (DOCX 13 kb) [file 262_2023_3404_MOESM6_ESM.docx]

**Supplementary Fig.1.** Overall survival (OS) by Child-Pugh score in the overall cohort and by Barcelona Clinic Liver Cancer (BCLC) stage. (**a**) OS by BCLC C vs. A/B; (**b**) OS by Child-Pugh A vs. B in patients with BCLC stage C; (**c**) OS by Child-Pugh A/B7 vs. B8-9 in the overall cohort; (**d**) OS by Child-Pugh A/B7 vs. B8-9 in patients with BCLC stage C; (**e**) OS by Child-Pugh A vs. B7 vs. B8-9 in the overall cohort; (**f**) OS by Child-Pugh A vs. B7 vs. B8-9 OS by Child-Pugh A vs. B7 vs. B8-9 in the overall cohort. OS: Overall survival; BCLC: Barcelona Clinic Liver Cancer.

**Supplementary Fig.2.** Overall survival (**a**) and progression-free survival (**b**) by subgroups in patients who received immune checkpoint inhibitors (ICI) monotherapy and ICI plus tyrosine kinase inhibitors (TKIs) [combination therapy](javascript:;) in the overall cohort.

**Supplementary Fig.3.** Overall survival (OS) and progression-free survival (PFS) in patients who received immune checkpoint inhibitors (ICI) monotherapy and ICI plus tyrosine kinase inhibitors (TKIs) [combination therapy](javascript:;) by Child-Pugh stage. **(a)** OS in Child-Pugh A; **(b)** PFS in Child-Pugh A; **(c)** OS in Child-Pugh B; **(d)** PFS in Child-Pugh B.

**Supplementary Fig.4.** Overall survival in patients with disease stabilization and progressive disease. (**a**) Total cohort, (**b**) Child-Pugh stage A, and (**c**) Child-Pugh stage B.

**Supplementary Table 1.** The [therapeutic regimen](javascript:;)s in seven patients with complete response.

**Supplementary Table 2.** Factors associated with 12-month overall survival and objective response rate in univariate analysis.

**Supplementary Table 3.** Factors associated with 12-month overall survival and objective response rate in multivariate analysis.
